# A qualitative study examining the critical differences in the experience of and response to formative feedback by undergraduate medical students in Japan and the UK

**DOI:** 10.1186/s12909-023-04257-6

**Published:** 2023-06-05

**Authors:** An Kozato, Kiyoshi Shikino, Yasushi Matsuyama, Mikio Hayashi, Satoshi Kondo, Shun Uchida, Maham Stanyon, Shoichi Ito

**Affiliations:** 1 Postgraduate Education Centre, Ipswich Hospital NHS Trust, Ipswich, UK; 2 Ipswich Hospital, Heath Rd, IP4 5PD Ipswich, UK; 3 Health Professional Development Center, Chiba University Hospital, Chiba, Japan; 4 Department of General Medicine, Chiba University Hospital, Chiba, Japan; 5 Medical Education Centre, Jichi Medical University, Tochigi, Japan; 6 Center for Medical Education, Kansai Medical University, Osaka, Japan; 7 Master of Medical Sciences in Medical Education, Harvard Medical School, Boston, MA USA; 8 Department of Medical Education Studies, Graduate School of Medicine, International Research Center for Medical Education, The University of Tokyo, Tokyo, Japan; 9 Center for Medical Education and Career Development, Graduate School of Medicine, University of Toyama, Toyama, Japan; 10 Center for Medical Education and Career Development, Fukushima Medical University, Fukushima, Japan; 11 Department of Medical Education, Graduate School of Medicine, Chiba University, Chiba, Japan

**Keywords:** Feedback, Formative assessment, Culture, Reflection, Constructivist grounded theory

## Abstract

**Background:**

Formative feedback plays a critical role in guiding learners to gain competence, serving as an opportunity for reflection and feedback on their learning progress and needs. Medical education in Japan has historically been dominated by a summative paradigm within assessment, as opposed to countries such as the UK where there are greater opportunities for formative feedback. How this difference affects students’ interaction with feedback has not been studied. We aim to explore the difference in students’ perception of feedback in Japan and the UK.

**Methods:**

The study is designed and analysed with a constructivist grounded theory lens. Medical students in Japan and the UK were interviewed on the topic of formative assessment and feedback they received during clinical placements. We undertook purposeful sampling and concurrent data collection. Data analysis through open and axial coding with iterative discussion among research group members was conducted to develop a theoretical framework.

**Results:**

Japanese students perceived feedback as a model answer provided by tutors which they should not critically question, which contrasted with the views of UK students. Japanese students viewed formative assessment as an opportunity to gauge whether they are achieving the pass mark, while UK students used the experience for reflective learning.

**Conclusions:**

The Japanese student experience of formative assessment and feedback supports the view that medical education and examination systems in Japan are focused on summative assessment, which operates alongside culturally derived social pressures including the expectation to correct mistakes. These findings provide new insights in supporting students to learn from formative feedback in both Japanese and UK contexts.

**Supplementary Information:**

The online version contains supplementary material available at 10.1186/s12909-023-04257-6.

## Background

### Feedback in medical education

Feedback is an integral process of the learning cycle during clinical placements [1, 2]. Ende [3] defined feedback in clinical medical education as ‘information describing student’s performance in a given activity that is intended to guide their future performance in that same or in a related activity’. Therefore, feedback can be a powerful tool in improving a learner’s performance [4].

Educators internationally are working towards an enhanced use of formative feedback [5]. The world federation for medical education (WFME) Global Standards for Education emphasises on-site observational assessment [6, 7], enhanced feedback through portfolios, and the promotion of learner growth through this process. These measures aim to transform the learning culture of clinical education by integrating feedback within the curriculum.

The educational effect of feedback is influenced by the contexts and cultural expectations formed by students and teachers [8]. When students expect feedback as a routine tool for learning, they accept its lessons more readily [9]. These internal landscapes have been developed in local contexts [10] at every organizational level – from personal to national levels. Local cultural context is acknowledged as a critical consideration for medical education [11], yet cultural nuances remain unexplored in detail [12].

Difficulty in deciphering the cultural influence on feedback is partly due to the complex and often ill-defined notion of culture. We employ an organizational perspective to define culture as ‘the collective programming of the mind’ that distinguishes a particular group [13, 14]. The collectives can be analysed on a different scale from macro (national and organizational) to micro (individual) level. Particularly, learning culture, implicated at organizational and national levels, can influence the context in which feedback is delivered by teachers and perceived by students.

### Learning culture – summative and formative paradigm

In clinical learning, student performance is observed during formative assessment and feedback provided. Subsequent improvement is evaluated at the end of the placement during summative assessment [1]. Formative assessment is a non-evaluative observation for correcting and improving students’ deficit in performing skills through feedback [15]. Furthermore, summative assessment is an evaluation of students’ performance to judge whether students have acquired the pre-determined learning outcomes and reached a desired threshold to proceed to the next stage of the curriculum. Formative and summative evaluations are distinguished by the way they are used, formative evaluations serve for future teaching and learning, and summative assessments serve as documentation of past learning [16, 17]. In clinical education, feedback and assessment are challenging for teachers to separate since they can exist simultaneously [18].

It is emphasised that feedback should be non-evaluative [19] as students’ reception of feedback is reduced when it is perceived in a summative context. For instance, students who received feedback based on performances in a summative objective structured clinical examination (OSCE) were more interested in the grade and did not fully read and accept the feedback comments [20]. Experience of frequent high-stakes assessment throughout a medical degree could lead to an orientation towards performance goals over reflective driven self-improvement [21]. Moreover, students may perceive receiving critical feedback as a ‘failure’ and develop feelings of ‘shame’ [20].

The marginalisation of feedback may be exacerbated by the learning culture of medicine [9]. Coaching relationships between students and teachers are often not facilitated due to pressures within clinical placements, where students rotate through various specialties without protected time for observation or regular feedback. Students may then use feedback in a goal-oriented way to pass the summative assessment at the end of the rotation on a short-term basis.

### Cultural influence on feedback in Japan and the UK

It is necessary to consider regional characteristics when implementing a global change. The educational effect of feedback has been drawn from studies based in English speaking regions, sometimes referred to as ‘Western’ countries [22, 23]. Numerous theoretical frameworks on formative feedback have been suggested, with evidence drawn largely from Anglophone contexts [24–26]. However, previous studies show that formative feedback is not always effective when applied to Asian contexts [27, 28]. Learners in Confucian heritage countries are thought to prioritise summative assessment at the expense of formative assessment [29, 30]. A previous study has identified the key elements within an assessment system that influenced receptivity to feedback emerging from different assessment cultures in Western countries [31]. However, they suggested to replicate this type of study in a different context to establish whether important themes regarding the uptake of feedback would be detected that they did not find. To date, no literature exists on how regional characteristics in assessment influence student experience of and engagement with formative feedback in Asian countries.

In this study, we examined how feedback was experienced by medical students in two countries – Japan and the UK - where students are subjected to different education and assessment settings. Both countries are geographically isolated islands, developing their educational structure with influences from Asian or European countries. Often, Japan is classified as an ‘Asian’ country and the UK as a ‘Western’ country, however simply using a binomial view would not be an accurate reflection of practice. We need to review the individual historical backgrounds of assessment and feedback in both contexts to examine how students experience feedback in clinical education.

Modern Japanese student ideas align with aspects of Confucianism, including a positive connection with good grades and social advancement and a belief in the usefulness of assessment. However, Japanese students do not show interest in intense competition and validation from family and book learning, which is stated as characteristics of ‘Asian’ students [12]. Japan’s geographical and political isolation prevented the establishment of the imperial examination system that had originated from China which spread to other East Asian countries [32].

Currently, the Japanese education system heavily relies on summative methods. Paper-based tests and multiple-choice questions remain the main format in traditional classrooms [33]. Furthermore, a large emphasis is placed on entrance examinations used for admissions to competitive and academically-focused high schools or universities [28, 34]. In Japan, medical students are high school graduates [35] who undertake four years of preclinical education. The preclinical phase includes liberal arts subjects and basic medical sciences [36], followed by two years of clinical education, where students participate in low to moderately invasive clinical activities [33]. Additionally, medical students on clinical rotations observe rather than actively participate in clinical care [37, 38].

The population make-up of the UK has developed to have high ethnic and linguistic diversity [39]. Compared to their Japanese counterparts, few studies have been conducted on UK medical students’ experience with and resulting attitudes towards assessment or feedback, possibly reflecting the difficulty of defining a constant characteristic of UK medical students as a singular group. The culture of clinical education has shifted to be learner-centred with a focus on the type of knowledge instead of the amount. As a result, communication skills, clinical skill, and continuous professional development are important outcomes for UK medical graduates [39].

In the UK, medical school admission comprises evaluation of grades, personal statements and interviews in most cases. Applicants are expected to provide qualitative evidence for their motivation and understanding of the medical career [40]. Additionally, medical students undergo two years of preclinical studies and three years of clinical medicine. The preclinical years include basic medical science subjects. Over the past three years, students have partaken in patient care under supervision [41]. Through clinical placement, final-year medical students are expected to gain competencies in clinical skills determined by the General Medical Council [42, 43]. Evaluation takes the form of workplace-based assessment, such as direct observation of patient contact, portfolio and multisource feedback from healthcare professionals [41]. Supervisors provide formative feedback by reviewing these components. Subsequently, students reflect on the formative assessment and feedback for their personal development and as evidence for a successful completion of the clinical years, before undertaking summative written and practical exams [42].

### Research question

This study examined how formative feedback whilst on clinical placement was experienced by medical students in two different cultures of feedback and assessment, and how this impacts their response to formative feedback. We selected medical students from Japan and the UK. We analysed how students received and reflected on teachers’ feedback during their clinical placement. We hypothesize that student reception to feedback is informed by the cultural context of previous educational environments. By exploring the students’ response to formative feedback, we aim to develop a theoretical relationship between the cultural characteristics of assessment and the student experience of formative feedback in a clinical setting. Specifically, we aim to examine:

Q1. Does the experience of feedback differ between students from Japan and the UK?

Q2. Does the student response to feedback exhibit influences of regional characteristics about assessment and feedback?

## Methods

### Study design

This study is of qualitative design set within a constructivist paradigm [44]. We followed the Standards for Reporting Qualitative Research recommendations [45]. Medical students’ experiences of and response to formative assessment were explored using semi-structured interviews. A constructivist grounded theory lens [46] was used to investigate differences in the medical student response to formative feedback between Japan and the UK. Due to little being known about the process of student perception of feedback in Japan and the UK, constructivist grounded theory was considered suitable to construct a theory on the influence of local assessment and feedback culture on student response to feedback [47, 48].

### Sampling methods and selection criteria

Theoretical sampling was carried out to strengthen theoretical sensitivity [49]. Medical students were recruited from one medical school in Chiba, Japan, and three medical schools in London, UK. These organisations were selected based on their accessibility to the researchers. Only students who had started clinical placements were recruited. The inclusion criteria were as follows: medical students currently on clinical clerkship, at least 6 months of clinical clerkship experience, experience with formative assessment during clinical practice, and students who have completed their higher education in either Japan or the UK. Recruitment was conducted from November 2019 to January 2021. Participants accessible to the researchers and fits the inclusion criteria were approached by KS and AK. This selection ensured a strategic sampling to theorise the process of feedback perception [41]. Participants were provided with a participant information sheet (Appendix 1) and consent form (Appendix 2). Moreover, they were also provided with the contact information of the lead researcher and a consent withdrawal form (Appendix 3). They were informed that they could withdraw from the study at any point, that identifiable information would be anonymised, and once the analysis was completed, personal data would be destroyed.

### Data collection

Prior to the interview, participants were requested to complete a questionnaire to collect their demographic data (Appendix 4). Semi-structured pilot interviews were conducted by AK and KS. YM, and SI were involved in revising the interview guide (Appendix 5). AK and KS re-interviewed participants after the revision. Questions used in the 20-minute semi-structured interviews were formulated to correspond to the research question. In the interview, participants were requested to recall their most recent experience of formative feedback. The experience was defined as a direct observation in a clinical environment followed by feedback with no summative weighting. Questions focused on participants’ emotional reactions towards their experiences and behavioural changes after the formative assessment. The format of the assessment was not defined in the question as assessment methods varied between the two countries. The UK student interviews were conducted by AK, a final-year medical student based in the UK. The Japanese student interviews were conducted by KS, an attending physician with experience in clinical medical education in Japan. The interviewers had experience in higher education in their respective countries and had previously conducted educational research. Due to the COVID-19 pandemic, interviews were conducted virtually on Zoom® as an alternative to face-to-face interviews [50]. Both video and audio were turned on during the interviews. Only the audio data were transcribed. Interview was conducted until the research team agreed meaning saturation was reached.

### Analysis

Data was analysed through a constructivist grounded theory lens [47]. A theory was derived with a focus on participants’ views, values, feelings and beliefs [46]. Textual data were coded openly and then axially to form abstract categories using a latent projective approach.

After each interview, AK and KS conducted the initial coding to explore emerging codes. No consensus or rule was set before the process to allow for open coding. The codes were refined and reviewed by YM based on the initial results. Before proceeding to the intermediate coding, AK, KS and YM agreed data saturation was reached, and no further code could be identified [41]. The intermediate coding was conducted by AK, KS and YM, in which a codebook was produced. Data were separated into indicators – small segments of information from raw data. They were assigned a property, dimension, and code. Property is defined as the subcategories of open codes to provide details of each category, whilst dimensions are features of the property in the continuum [46]. Results were shared with the rest of the research group (SK, MH and SU). Each member then analysed the data from at least one UK and one Japanese student. The Japanese data and the English data were analysed without translation. After the data were coded, researchers discussed the results with AK conducting axial coding based on this discussion. Axial coding is defined as the process of categorize intermediate coded units and revealing the relationship between categories. Coded units were grouped into overarching categories to form a theoretical framework for each student group. The framework was structured into a coding paradigm, formed by the core categories (students’ perception of feedback) and the relationship between the contextual categories (students’ understanding of the context and environment) and consequential categories (students’ emotional response and changes in learning approach). These results were shared among the research group for further discussion. AK and KS performed one final interview in each cohort. The resultant codes were reviewed by the research team to confirm no further information was emerging to form new categories and meaning saturation was achieved [51].

Advanced coding was conducted by AK with a storyline technique utilised to further develop the overarching themes, which were reviewed by YM, KS and HM, and interpreted within a grounded theory paradigm. This was reviewed by SK, SI and SU.

MS, a medical education researcher with experience in UK medical education and currently working in Japan provided critical oversight of the results and discussion. This final stage served to integrate an etic perspective, as the research team largely consisted of members with education experience based in Japan.

### Researcher reflexivity

At the time of the study, AK, who primarily analysed the data, was a medical student with a background of secondary education in both Japan and the UK. She has experienced a Japanese summative examination system up to secondary education and then transferred to the UK for higher education (high school and university). University admission through a personal statement and interview process introduced the need for formative feedback as a newly learnt concept, which may influence the interpretation of the students’ perceptions from her views as both an insider and outsider in both cohorts.

MS was the only other researcher with experience of medical education teaching in both Japanese and UK contexts. The rest of the research team are educators working in Japan. As undergraduates themselves, they experienced a curriculum dominated by summative feedback, and now are involved in the supervision of students and curriculum development as education faculty. Their viewpoints as educators in Japanese medical education may influence their perceptions for potential improvement in Japan, and support feelings towards UK practice as a model example.

## Results

Eleven Japanese and thirteen UK students were recruited. Both groups included male and female students who had already completed 1–3 years of clinical placement. The age range of participants was 22–25 years (Table 1). The Japanese students had all completed their previous education in Japan and entered medical school immediately after graduating from high school. In the UK cohort, 1 student obtained a degree prior to entering medicine. 11 students completed intercalated degrees during their medical courses. 3 students received primary education in Nepal, Iran and Norway respectively. 2 students received part of their secondary education in Japan and France.

**Table 1:** Participant demographic data Legend: Thirteen UK students and eleven Japanese students were recruited. Both cohorts included male and female students, aged between 22 and 25. All students were in their clinical years. Twelve students in the UK cohort held an additional degree

	Age	Gender	Course year	Years spent in clinical placement	Additional degree
UK	22	F	4	1–2 years	iBSc
	23	F	4	1–2 years	iBSc
	25	M	4	1–2 years	BSc
	22	F	4	1–2 years	iBSc
	22	M	4	1–2 years	iBSc
	22	M	4	1–2 years	iBSc
	22	F	4	1–2 years	iBSc
	22	M	4	1–2 years	iBSc
	22	M	4	1–2 years	iBSc
	22	F	4	Less than 1 year	No
	25	F	5	2–3 years	iBSc
	22	F	4	1–2 years	iBSc
	23	F	4	1–2 years	iBSc
JAPAN	24	M	5	Less than 1 year	No
	23	M	5	Less than 1 year	No
	23	M	5	Less than 1 year	No
	25	M	5	Less than 1 year	No
	23	M	5	Less than 1 year	No
	24	F	5	Less than 1 year	No
	24	F	5	Less than 1 year	No
	23	M	5	Less than 1 year	No
	23	M	5	Less than 1 year	No
	23	M	4	Less than 1 year	No
	22	M	5	Less than 1 year	No

Following a constructivist grounded theory approach, we explored the student viewpoints towards formative assessment and how students dealt with the feedback received. The setting in which feedback was given differed between the two cohorts. Two themes emerged from our analysis of students’ response to and experience of formative feedback during their clinical placements. Although two cohorts interpreted the purpose of formative feedback differently as discussed below, all students in general held a belief that their experiences helped them to become competent future practitioners.

### Feedback setting

Among Japanese students, feedback was given face to face, immediately after patient contact. In the UK, feedback was delivered while students were involved in clinical care of the patients. The observation was usually on an ad-hoc, unplanned basis. Japanese students prepared in advance for the session, which was seen as a nerve-wracking time. This was described by two students as follows:

“*I will face that time [formative assessment] with the pressure of understanding that I am being assessed. The learning in preparation was very useful for me. … Yes, I think there was a nervousness beforehand. Because I was nervous, I did prepare for it*”

#### (J3-7 ~ 10)

“*I was in a care home so [I] just asked to speak to a patient who had dementia and basically assessed how he was at the time, because he had been discharged from the hospital quite recently. … I was taking part in some activities in their care as well…It really wasn’t anything like a formal examination. And so, the GP was in the room at the time … And so, I got feedback as I was doing it, rather than at the end.*”

#### (U3-1)

### Theme1: experience of feedback as a model answer or an opinion

Japanese students treated the feedback as an answer that should be modelled. They emphasized how the desirable clinical skills and attitudes are not available from textbooks or lectures. Due to the unavailability of answers, they lacked confidence in whether they were dealing with the clinical problems in a ‘correct’ way, and thus considered the feedback to be invaluable. Once they were provided with the feedback, it was used to identify and reduce the gap between their performance and that of the experts. The Japanese verb ‘kaizen’ was frequently seen, which describes the reflective action of identifying an error and making a correction.

“*How I speak to patients whilst taking a history cannot be assessed in lectures or normal lessons, [it is] a rare opportunity. There was no example that I could think of, [about] what I should be doing. As a result, I started observing doctors’ interaction with patients more carefully.*”

#### (J3-14)

“*After the history taking, we went back to the conference room. I had feedback on the history and how to examine a patient … Things I thought I did alright with were pointed out to be not done well. It was a very useful [exercise] as I saw room for kaizen*”

#### (J4-1)

This was in contrast with the UK students. Feedback was an opinion rather than an answer, which was used as a material to establish their own desired goal. Feedback was part of the experiential learning, and not an endpoint of formative assessment.

“*…that really stuck with me because it wasn’t like ‘This is how you do it.’ It was more akin to ‘This is how I do it. How would you do it? You should go away and reflect on this and return. I’d be really interested in seeing what you came up with’…*”

#### (U13-5)

Accepting and following the feedback was a default reaction among the Japanese students. One Japanese student described how modelling feedback produced inconsistent results when he was assessed in another placements. In anticipation of a tutor disapproving of the modelling and reproduction of behaviours led to a reluctance in being observed in a different speciality. UK students in contrast were more selective and made distinctions between constructive and non-constructive feedback, only digesting selected feedback.

“*The feedback given by different tutors is not consistent. At one time a behaviour was seen as good but not in another specialty. This led me to consider what the tutor in the current placement thinks before I make the move. In that sense, I think feedback becomes a sort of a burden*”

#### (J8-21)

*“… based on the previous experiences of not having been acknowledged for answers that I gave that were correct but which were dismissed by the GP as incorrect, I suppose in my future approaches with that particular individual, I [will] place less emphasis on the learning that I got from there. I just tried to focus on small, self-directed learning or from clinical teachers in the practice… ”*.

#### (U3-10)

Students in both cohorts reflected on the formative feedback. There was a difference in how the reflective cycle progressed. The aims of Japanese students were to meet tutor expectations, whereas UK students established their own position against that of tutors.

### Theme 2: response to feedback as an outcome or a material for judgement

As discussed above, Japanese students treated the feedback given as a model answer. They viewed the feedback as a judgement of their performance, and the formative assessment as an opportunity to gauge whether they are achieving a standard expected by the tutors. When the tutor granted affirmation to students it invoked a positive emotional response of relief and satisfaction. The focus on performing satisfactorily was also illustrated in students’ embarrassment to being compared to their peers.

“*… I was not confident in taking the history. When my tutor said he agreed with how I used some words when trying to be empathetic towards a patient, I was able to confirm that what I was thinking and doing was right. I was glad.*”

#### (J7-3)

“*If I am being observed in front of everyone and not one to one, I would wish for someone smart to do the task, not me. I think people not doing well, including me, would want that. We should try to avoid the embarrassment*”

#### (J1-14)

For UK students, formative feedback was not interpreted as a concrete judgement. While the feedback involved comments on how well tutors felt students performed, it was often seen as an opinion. Although they also used the feedback given to identify the lack of knowledge or skills, the reflective scaffolding process the feedback was based on was seen as the fundamental purpose of the formative assessment.

“*… It wasn’t just something, you know, as you walked out of the room, that was like, “Yeah, that was good”. It was like sat down, and then they asked, you know, what I thought went well, what I thought went wrong and kind of like self-reflection, … and kind of giving you the space and time to reflect on what that meant with them, you know.”*

#### (U6-9)

### Theoretical framework: the goal of formative feedback is external validation for japanese students and internal scaffolding for UK students

The two themes show characteristic differences in the experience and response to formative feedback. as a model answer provided by teachers, focusing on the collection of ‘tips’ from experts without selection, which is not available from pre-clinical materials, such as lectures. They tended to see feedback as an invaluable source of wisdom and employed modelling behaviours to reproduce the examples seen. Feedback was seen as a judgement on whether they were fulfilling the teacher’s expectation, which was treated as the required standard. In response to feedback, Japanese students targeted their efforts on how they could meet external expectations. UK students in contrast interpreted feedback as an opinion of their teachers. They measured their own opinion of their performance in relation to the feedback given before accepting the feedback, with the goal of understanding what their performance meant for them in relation to their own values.

## Discussion

Although Japanese students in this study were interviewed exclusively about formative assessments, they still focused on whether their performance met summative standards, with feedback used as a surrogate pass/fail marker. By comparison, UK students focused on how the feedback could benefit their personal development. For them, the feedback was an opinion that may or may not aid their learning. They did not focus on whether they had satisfied the tutor’s expectations or viewpoint .

This difference may be a reflection of the assessment paradigm operating in each country. The belief that scoring well on the university entrance examination and that being admitted to a university which requires a high entrance score would ensure a stable future is relatively strong in Japan [52, 53]. For this reason, students are sent to cram schools and preparatory schools in addition to the regular school curriculum in order to prepare for university entrance examinations [30]. A high test score is required to enter a medical school, and medical school applicants recognize that the way to pass the examination is to be thoroughly trained in strategies and faithfully follow the teachings of charismatic teachers [12, 54]. In the UK, medical schools assess students qualitatively through interviews and personal statements as part of the admission process alongside their exam grades [41]. Students are required to express their own opinions and values instead of following instructions in open evaluations. This orientation towards self-expression and value formation by the UK students was observed in the study.

Japanese students interpreted the feedback received as model answers and attempted to correct their performance to replicate expert behaviour. They reflected on what could be modified in order to meet tutor expectations in the future. The feedback was accepted in a non-selective manner compared to the UK students. UK students viewed the feedback as material for their experiential learning. A unidirectional delivery of feedback was readily welcomed by Japanese students, whereas UK students appreciated bidirectional discussion and questioning.

Responses to feedback may originate from the high-power distance in student-teacher relationship observed in east Asian countries [13, 55]. Students are expected to follow the social script according to the standard shown by the tutors without question [56]. In this context, students naturally follow the social rules of accepting feedback in a non-selective way. Furthermore, silence is regarded as a collaborative practice to handle conflicting understandings by Asian students, including Japanese [57, 58]. It is possible that Japanese students do not identify questioning and discussing feedback as a learning strategy. The UK is a low-power distance society which permits the cultivation of ground for discussions and the egalitarian formation of educational alliances. This is a framework in which students recognise the tutors’ commitment to student progress, which fosters effective feedback [59] and the building of effective and trusting relationships. UK students may therefore be more perceptive of the dynamic of the relationship as part of the feedback process when compared to their Japanese counterparts.

It is important to note the diverse educational and cultural background present in the UK cohort when compared to Japanese cohort. Exposure to different values as well as the transfer between different cultural environments may lead students to perceive values ascribed to the feedback in more fluid and relativistic ways. Japanese students who move into other mono-ethnic systems may find the more absolute and concrete values associated with feedback to be more consistent with their previous experience.

### Implications

The experience of and response to feedback in an exclusively summative way by Japanese students poses a challenge to facilitating a culture of ‘assessment for learning’ [20]. Formative assessment at medical school is intended to foster learning and encourage reflection and self-regulated learning [60]. Labelling the formative assessment as pass/fail activity and the delivery of feedback as a concrete answer illustrates how summative elements remain strong within some cultural contexts. Carless [52] acknowledged this challenge and suggested the Formative Use of Summative Test (FUST), where students are guided through a self-reflective process based on summative results. A study in an Indonesian medical school found that feedback given from summative assessments enhanced the learning effect [19]. The data suggests an interesting compromise of educational principle in the face of local context.

It is important to note that Japanese students’ tendency to copy the tutor’s feedback as an absolute answer may not always be detrimental. In fact, it could be a useful characteristic in short-term skill acquisition and fulfilling their role as a junior member. A study conducted in the US found the residents did not accept the feedback depending on the sender credibility and delivery manner [61]. Similar results were observed in our study among the UK students. Asian students focus on following feedback could be considered as an example of a positive dispersion of power, especially in the context of teamworking, in which a strong structural working relationship ensures smooth decision making and good standard of care [62].

‘Hansei’ is a Japanese concept that means critical reviewing and evaluation of past behaviour in order to finally improve upon it. The habit of hansei is seen as a fundamental skill for social development in Japanese society. The concept emphasizes the importance of identifying a negative point so that changes can be made. This cycle is known as ‘kaizen’, which is an ingrained aspect of Japanese society [10]. Focus on accepting their own mistake from a leader’s feedback and changing the error could strengthen organizational drive to change for the better [63]. In a healthcare setting within this context, student behaviour of non-critical acceptance of feedback may be beneficial to patient care and safety.

However, clinicians are expected to continue life-long professional learning beyond their junior years, where simple modelling of senior figures would not be sustainable. While recognising the role of summative assessment discourse, students should be facilitated with self-reflective learning and continuous professional development [54] from the early years of their career.

Another important stakeholder in formative assessment is the clinical tutor. Effective feedback requires an appropriate prompt and initial guidance on their part. We have not explored this in detail. However, given the tendency of Japanese students to model the tutors’ feedback, we feel it would be a good starting point for tutors to actively encourage students to engage in two-way conversation during feedback. A longitudinal curriculum has been shown to be successful in establishing self-reflective learning, due to the facilitation of a stronger tutor-student relationship [22]. In addition, many Japanese clinical tutors themselves were exposed to educational contexts with a heavy weighting on summative assessment as students, therefore the training of the tutors in feedback given in formative contexts should be a priority.

## Limitations

This study has some limitations. The interviews were conducted by AK for UK students and KS for Japanese students. AK and KS had different relationship dynamics with students due to one being a peer and the other being a supervisor. Therefore, it is possible that the interaction between the interviewer-interviewee relationships may have been influenced in different ways. Both AK and KS had trusting relationships with the interviewees, which was favourable for study credibility.

Moreover, this study was not longitudinal. Continuing to follow participants during their clinical clerkship would allow us to track the development of their personal identities and provide deeper insight into how they utilised their individual experiences.

Due to resource availability, only students from three medical schools in London and one medical school in Japan were recruited. Therefore, given the importance of context in professional identity formation, the results may not be transferable. This point may not have solidified the triangulation of collecting multiple data about student’s perception. In addition, the small number of participants may indicate that the conceptual framework is based on unique student experiences and may not be generalizable. Further research in other contexts is needed to examine the transferability of these findings.

## Conclusions

This study showed students in Japan and the UK experienced and responded to feedback differently reflecting key contextual differences. Our data highlights the influence of the assessment paradigm operating within each country and the impact on expectations and reactions to feedback. The goal of feedback differed between the cohorts. In a Japanese setting, feedback was seen as an answer that the student must model, with Japanese students trying to identify what they lack from the viewpoint of the assessors and altering their practice to meet tutor expectation. UK students utilized feedback selectively to aid self-reflection. As a result, the purposes ascribed to the feedback process may reflect different cultural expectations on the student-tutor relationship. Further qualitative research into the perception of tutors would be useful in understanding this phenomenon. To successfully implement formative assessment to enhance learning, the sensitive implementation of curriculum design to facilitate culturally appropriate formative feedback and educator training compatible with the local context could play a significant role.

## Electronic supplementary material

Below is the link to the electronic supplementary material.

Supplementary Material 1

Supplementary Material 2

Supplementary Material 3

Supplementary Material 4

Supplementary Material 5

## Data Availability

The datasets obtained and analysed during the current study can be availed through the corresponding author upon reasonable request.

## Abbreviations

GMC: General Medical Council
OSCE: Objective Structured Clinical Examination
WFME: World Federation for Medical Education
