# Supplementary material for: A qualitative study examining the critical differences in the experience of and response to formative feedback by undergraduate medical students in Japan and the UK
Source: BMC Med Educ. 2023 Jun 5;23:408. doi: 10.1186/s12909-023-04257-6 (PMC10240445; doi:10.1186/s12909-023-04257-6)
Supplement: Supplementary file 1 — Supplementary Material 1 [file 12909_2023_4257_MOESM1_ESM.docx]

Appendix 1. Participant information sheet

Cultural influence on medical students' perspectives on assessment in placement in Japan and the UK

Participant information sheet

This document contains the information about the research study you are invited to participate, including the nature of the study and details of the consent to participate. This research was approved by the Ethics committee at Chiba University Graduate School of Medicine and Student Project Assessment Committee of Queen Mary University of London to ensure the ethical acceptability including the protection of human rights. If you would like to participate in this study after carefully reading this sheet, please sign the consent form below. You would never be disadvantaged by deciding not to participate.

If there is anything unclear or you would like to receive more explanation, please do not hesitate to contact the researcher.

1. The purpose of the study

This study aims to produce a socio-culturally diverse picture of how the assessments in clinical placements are perceived by students and teachers. By identifying the differences between the two countries, we want to establish how cultural and historical factors hinder/promote assessments. The data derived will be applied to provide clinical education with improved quality assessment and feedback to benefit the medical students, teachers and wider education community.

1. Research method and timeframe

1) Data to be collected and the collection method

In this research, we would collect demographic information of the participants via questionnaires. We would then like to interview the participants individually to generate qualitative data regarding the participants’ perceptions on the current assessment in clinical education. The data will be collected and analysed. The data will be collected and stored by Chiba University Graduate School of Medicine for the purpose of institutional research.

2) Data storage site

The data would be stored by the research within Chiba University Graduate School of Medicine.

3) Data storage method

The data obtained in this research will be stored by the researcher. Data will be anonymized and assigned ID. They are stored only by the researcher in charge, and even other co-researchers do not have access to the data before anonymization.

4) Research timeframe and schedule

This research will be carried out until June 2021. During this period, data collection will be held.

1. Anticipated benefits

This study aims to provide better understanding into the assessment process in the current clinical education in the international context. Better understanding of cultural factors would advise educators how to create effective assessment of an increasingly diverse medical student population.

1. Anticipated risks

This study will be carried out in the form of questionnaires and interview. The contents will not contain sensitive subjects. Therefore, risk causing physical or psychological harm to the participants is not anticipated at all.

1. No disadvantage for non-participation

You will never be disadvantage by not agreeing to participate in this study. The participation is voluntary.

1. Rights to withdraw

You are entitled to withdraw from the study after consenting to participate. If you withdraw, corresponding data will be destroyed.

1. Handling of personal information

Any information provided will be stored upon being anonymised to unable personal identification. Thus, your personal information will never be leaked externally.

The outcome of this study could be published in academic journals or conferences. Your information will again never be made public in these occasions.
