# Supplementary material for: A qualitative study examining the critical differences in the experience of and response to formative feedback by undergraduate medical students in Japan and the UK
Source: BMC Med Educ. 2023 Jun 5;23:408. doi: 10.1186/s12909-023-04257-6 (PMC10240445; doi:10.1186/s12909-023-04257-6)
Supplement: Supplementary file 2 — Supplementary Material 2 [file 12909_2023_4257_MOESM2_ESM.docx]

Appendix 2. Study consent form

Consent Form

I confirm that I have received and understood the verbal and written explanation about the study, “Cultural influence on medical students' perspectives on assessment in placement in Japan and the UK”, including its purpose and procedures, and agreed to the points listed below. I hereby confirm that I have voluntarily agreed to participate in this study.

Explained and understood points (Please tick)

Purpose and importance of the study

Research methods

Anticipated benefits

Anticipated risks

No possibility of disadvantages in the event of not agreeing to participate

Right to withdraw the consent after initially giving consent to participate

Personal information handling

Researcher details and contacts

Participant’s signature

                                                                                            Date
