# Supplementary material for: A qualitative study examining the critical differences in the experience of and response to formative feedback by undergraduate medical students in Japan and the UK
Source: BMC Med Educ. 2023 Jun 5;23:408. doi: 10.1186/s12909-023-04257-6 (PMC10240445; doi:10.1186/s12909-023-04257-6)
Supplement: Supplementary file 3 — Supplementary Material 3 [file 12909_2023_4257_MOESM3_ESM.docx]

Appendix 3. Consent withdrawal form

Medical students' perceptions of assessment in clinical placement: a comparative study between the UK and Japan

Consent withdrawal form

I withdraw my consent to participate and agreements to the points listed below. Please do not use and store my data.

Please tick the points that apply.

(If you only tick points 1, it will still be regarded as you withdraw your agreement on point 2. If you only tick point 2, the data will be destroyed after the completion of this study.)

1. Agreement for the data to be used in this study

2. Agreement for the data to be used in the study in the future

Signature

                                                                                      Date
