# Supplementary material for: A qualitative study examining the critical differences in the experience of and response to formative feedback by undergraduate medical students in Japan and the UK
Source: BMC Med Educ. 2023 Jun 5;23:408. doi: 10.1186/s12909-023-04257-6 (PMC10240445; doi:10.1186/s12909-023-04257-6)
Supplement: Supplementary file 4 — Supplementary Material 4 [file 12909_2023_4257_MOESM4_ESM.docx]

Appendix 4. Pre-interview questionnaire

Cultural influence on medical students' perspectives on assessment in placement in Japan and the UK

Participant survey

1. Please state your age.

1. In which country did you receive
2. Primary education ?: _____________________________________________________________
3. Secondary education ?:   __________________________________________________________
4. Tertiary education ? (if applicable): _________________________________________________

1. Which medical school do you attend?

1. What year of your course are you currently in?

1 2 3 4 5 6  other (please state)_______________________________________________________

1. How many years of clinical placement have you had?

Less than 1 year / 1-2 years / 2-3 years / more than 3 years
