# Supplementary material for: A qualitative study examining the critical differences in the experience of and response to formative feedback by undergraduate medical students in Japan and the UK
Source: BMC Med Educ. 2023 Jun 5;23:408. doi: 10.1186/s12909-023-04257-6 (PMC10240445; doi:10.1186/s12909-023-04257-6)
Supplement: Supplementary file 5 — Supplementary Material 5 [file 12909_2023_4257_MOESM5_ESM.docx]

Appendix 5. Interview guide

Q1. Please remember the most recent experience when your patient contact was directly observed and assessed. Can you describe that experience briefly including the feedback that followed during the clinical placement?

Q2.　How did you feel when you received the feedback you mentioned in Q1?

Q3.　What change did the feedback have on how you approach learning? Please explain both positive and negative change and the reason.
